# Kentucky

**Published:** 1867-07

**Authors:** 


### EDITORIAL AND MISCELLANEOUS

---

### KENTUCKY.

*Death of Dr. Miller, Dr. Linthicum, and Dr. Cosby.*

We are again at our post, having spent three months in dear old Kentucky, where, after an absence of six years, the noblest hospitalities were extended to us. Every where we met old friends, and every where the most refreshing assurances of good will. God bless the noble old State and her glorious people. But amid the rejoicing incident to a hundred re-unions, there were also causes of sadness. Not a few of our dear old professional brothers and fathers have crossed the shadowy river, and gone to that "bourne from whence no traveler returns." Dr. Wm. Miller, of Madisonville, Kentucky, our friend and preceptor, a man of talents and ability, and who first taught us how to think, died in 1863, aged sixty-nine years. He was a christian gentleman, born and educated in Virginia, but spent his whole professional life in Kentucky, where he ranked among his peers as a medical philosopher of rare attainments. Dr. Rufus K. Linthicum, of Henderson county, Kentucky, our dear friend, and for seven years our partner in the practice of medicine, died in the beginning of 1864. He was a man of marked ability, and so ranked amongst his brethren of the profession—aged fifty-five years. Dr. Garland Cosby, also of Henderson county, Kentucky, died on the 5th of November last. Born in Kentucky, and raised an orphan, without friends or money, he won his way to distinction by his talents and energy alone. He died full of years and full of honors, aged seventy-six. Each of these gentlemen leave families, and each family has its representative of acknowledged talent and ability. If our earnest good wishes could benefit them, much of the earth and its fullness should be theirs, and none of the moral worth of their excellent fathers lost.

